# Corticosteroid treatment for community-acquired pneumonia - the STEP trial: study protocol for a randomized controlled trial

**DOI:** 10.1186/1745-6215-15-257

**Published:** 2014-06-28

**Authors:** Claudine A Blum, Nicole Nigro, Bettina Winzeler, Isabelle Suter-Widmer, Philipp Schuetz, Matthias Briel, Roland Bingisser, Werner Zimmerli, Elke Ullmer, Hanno Elsaesser, Philip Tarr, Sebastian Wirz, Robert Thomann, Eveline Hofmann, Nicolas Rodondi, Hervé Duplain, Dieter Burki, Beat Mueller, Mirjam Christ-Crain

**Affiliations:** 1 Endocrinology, Diabetology and Metabolism, Department of Internal Medicine, University Hospital Basel, Petersgraben 4, 4031 Basel, Switzerland; 2 Medical University Clinic, Kantonsspital Aarau, Tellstrasse, 5001 Aarau, Switzerland; 3 Basel Institute for Clinical Epidemiology and Biostatistics, University Hospital Basel, Hebelstrasse 10, 4031 Basel, Switzerland; 4 Department of Clinical Epidemiology and Biostatistics, McMaster University, Hamilton, ON, Canada; 5 Emergency Department, University Hospital Basel, Petersgraben 4, 4031 Basel, Switzerland; 6 Medical University Clinic, Kantonsspital Baselland Standort Liestal, Rheinstrasse 26, 4410 Liestal, Switzerland; 7 Medical University Clinic, Kantonsspital Baselland Standort Bruderholz, 4101 Bruderholz, Switzerland; 8 Clinic of Internal Medicine, Bürgerspital, Schöngrünstrasse 42, 4500 Solothurn, Switzerland; 9 Department of General Internal Medicine, Inselspital, Bern University Hospital, 3010 Bern, Switzerland; 10 Medical Clinic, Hôpital du Jura, Site de Delémont, Faubourg des Capucins 30, 2800 Delémont, Switzerland; 11 Viollier AG, Postfach, 4002 Basel, Switzerland

**Keywords:** Corticosteroids, Community-acquired pneumonia, Pulmonary infection, Emergency medicine, Intensive care, Glucocorticoids

## Abstract

**Background:**

Community-acquired pneumonia (CAP) is the third-leading infectious cause of death worldwide. The standard treatment of CAP has not changed for the past fifty years and its mortality and morbidity remain high despite adequate antimicrobial treatment. Systemic corticosteroids have anti-inflammatory effects and are therefore discussed as adjunct treatment for CAP. Available studies show controversial results, and the question about benefits and harms of adjunct corticosteroid therapy has not been conclusively resolved, particularly in the non-critical care setting.

**Methods/Design:**

This randomized multicenter study compares a treatment with 7 days of prednisone 50 mg with placebo in adult patients hospitalized with CAP independent of severity. Patients are screened and enrolled within the first 36 hours of presentation after written informed consent is obtained. The primary endpoint will be time to clinical stability, which is assessed every 12 hours during hospitalization. Secondary endpoints will be, among others, all-cause mortality within 30 and 180 days, ICU stay, duration of antibiotic treatment, disease activity scores, side effects and complications, value of adrenal function testing and prognostic hormonal and inflammatory biomarkers to predict outcome and treatment response to corticosteroids. Eight hundred included patients will provide an 85% power for the intention-to-treat analysis of the primary endpoint.

**Discussion:**

This largest to date double-blind placebo-controlled multicenter trial investigates the effect of adjunct glucocorticoids in 800 patients with CAP requiring hospitalization. It aims to give conclusive answers about benefits and risks of corticosteroid treatment in CAP. The inclusion of less severe CAP patients will be expected to lead to a relatively low mortality rate and survival benefit might not be shown. However, our study has adequate power for the clinically relevant endpoint of clinical stability. Due to discontinuing glucocorticoids without tapering after seven days, we limit duration of glucocorticoid exposition, which may reduce possible side effects.

**Trial registration:**

7 September 2009 on ClinicalTrials.gov: NCT00973154.

## Background

Respiratory infections, consisting mainly of pneumonia, are the third leading infectious cause of death worldwide [1]. Community-acquired pneumonia (CAP) is the main cause for sepsis and septic shock, both of which carry a high risk of long-term morbidity and mortality [2,3].

The standard treatment of CAP has not changed for the past 50 years, and its mortality and morbidity has remained high at 5 to 15% despite the availability of broad-spectrum antibiotics [4]. Systemic corticosteroids have anti-inflammatory effects [5,6] which, in CAP, attenuate the systemic inflammatory process which may have a detrimental impact [7] on the lung [8]. Therefore, adjunct treatment with corticosteroids has been discussed since the 1950s [9]. As early as 1955, favorable effects of corticosteroids were reported in patients with pneumococcal pneumonia [9]. A small-sized multicenter randomized trial showed a significant reduction in hospital mortality in severe CAP with a seven-day continuous infusion of hydrocortisone (240 mg/day), although this study was not powered for mortality [10]. Another large, but retrospective, single center study evaluated more than 300 patients and demonstrated in multivariable analysis that use of corticosteroids was associated with a lower mortality [11]. More recently, two randomized placebo-controlled trials involving 200 to 300 patients showed controversial results, one study showing no benefit of adjunct prednisolone treatment and one study showing a significant reduction in duration of hospital stay using dexamethasone [6,12]. Three systematic reviews [13-15] and one meta-analysis [16] concluded that the available evidence suggests that administration of corticosteroids in patients with CAP might be beneficial. However, a large and adequately powered randomized trial is warranted to support or refute this beneficial effect before recommendations can be made with regard to use of corticosteroids in the treatment of CAP. In addition, as the potential harmful effects of corticosteroids in high-dose and long-term treatment are well-known [17,18], special attention has to be given to the side effects of corticosteroid treatment.

## Methods

### Objective and design

The objective of this trial is to evaluate whether treatment with prednisone for seven days in patients with CAP as compared to placebo reduces time to clinical stability.

This is a multicenter randomized placebo-controlled trial. The local ethics committees: Ethikkommission beider Basel EKBB (Reference Number 370/08), Ethikkommission der Kantone Aargau und Solothurn (Reference Number 2011/031), commission d’ethique Lausanne (Reference Number 116/10) and Ethikkommission Bern (Reference Number 193/11), as well as the Swiss Agency for Therapeutic Products SWISSMEDIC (Reference Number 2009DR3227) approved the study protocol after meeting imposed conditions. This trial adheres to the declaration of Helsinki and was registered at clinicaltrials.gov as NCT00973154 on 7 September 2009. The protocol follows the recommendations of the SPIRIT initiative [19], and the trial results will be reported according to the latest version of the CONSORT statement [20].

### Study setting and participants

All adult patients with CAP are screened and enrolled at emergency departments or medical wards in seven tertiary care hospitals of Switzerland within 24 hours; on weekends within 36 hours of hospitalization. Eligible patients are asked for their informed consent and are then centrally randomized to either 50 mg prednisone or to placebo, taken orally for 7 days in a ratio of 1:1. If the patient is not able to give informed consent at the time of hospitalization due to severity of CAP, informed consent from next of kin and an independent physician is obtained. In these cases, informed consent from the patient himself has to be obtained as soon as possible. Patient recruitment started in December 2009 and is planned to be completed in April 2014.

Inclusion criteria are age of 18 years or older and hospitalization with CAP defined by a new infiltrate on chest radiograph and the presence of one or several of the following acute respiratory signs or symptoms: cough, sputum production, dyspnea, core body temperature ≥ 38.0°C, auscultatory findings of abnormal breath sounds and rales and leukocyte count > 10 or < 4 G/L [21].

Exclusion criteria are permanent inability for informed consent, active intravenous drug abuse, acute burn injury, gastrointestinal bleeding within the past three months, known adrenal insufficiency, a condition requiring more than 0.5 mg/kg/day prednisone equivalent, pregnancy or breast feeding, and severe immunosuppression defined as previously known infection with HIV and a CD4 cell count below 350 G/L, immunosuppressive therapy after solid organ transplantation, neutropenia < 500 G/L or a neutrophil count of 500 to 1,000 G/L with an expected decrease to values < 500 G/L in patients under ongoing chemotherapy, cystic fibrosis and active tuberculosis.

### Randomization and blinding

Allocation of patients to either intervention in this randomized placebo-controlled trial is concealed due to a pre-specified computer-generated randomization list which is kept centrally at the pharmacy of the main study center. Randomization between the two arms are 1:1 with variable block sizes of 4 to 6 and are stratified at the time of study entry by study center.

Generator and executor of randomization are separated. Patients are randomly assigned to receive a prepared set of study medication that contains 7 tablets of 50 mg prednisone or placebo. The drugs are prepared prior to the initiation of the study and packed by the Pharmacology Department, University Hospital, Basel, according to the randomization list. The numbered study medications are then delivered to the study team. By this method, study team including data analysts, patients and treating physicians will be blinded to group allocation. Unblinding will be performed only upon request of a treating physician or of the data safety and monitoring board (DSMB).

### Study protocol

**Figure 1:**
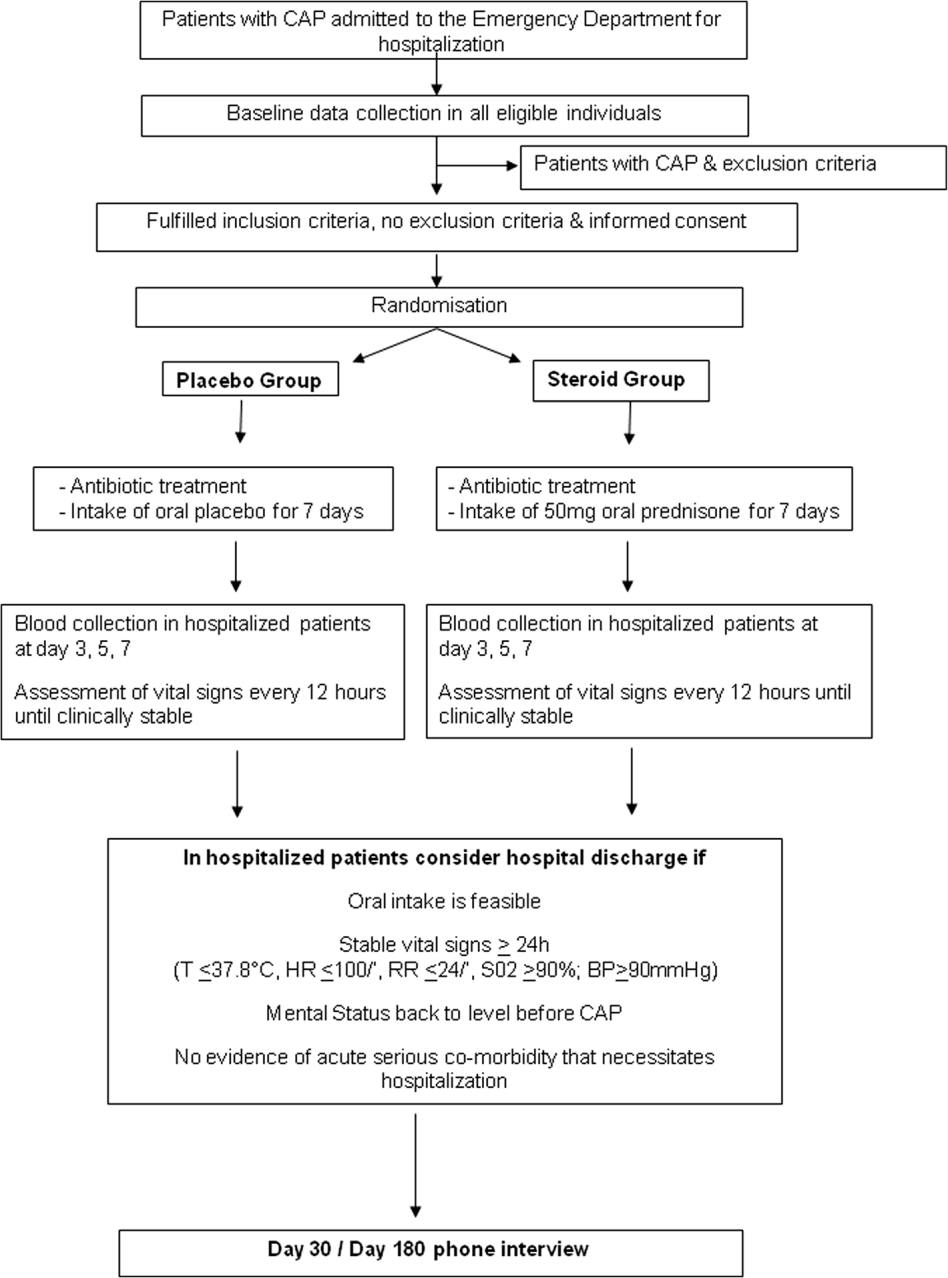
Study flow chart.

The study flow chart is shown in Figure 1. After informed consent is obtained, baseline blood is drawn and a low-dose adrenocorticotropic hormone (ACTH) test is performed (measurement of basal cortisol followed by cortisol after 0.001 mg of tetracosactid (Synacthen®)). Study medication is started after the ACTH test, and timing in relation to start of antibiotics will be monitored. Patients are evaluated for the primary endpoint of clinical stability every 12 hours during the hospitalization. All patients are being treated according to current CAP guidelines [22,23]. Antibiotic therapy guided by an established procalcitonin algorithm [24] is encouraged. The time of discharge is not determined by the study investigators, but based on the decision of the treating physician team.

### Laboratory assessment

The blood samples drawn on days 1, 3, 5, 7 and at discharge are immediately centrifuged and stored at -70°C until further processing. All laboratories of the participating centers are internationally validated and standardized. Cortisol is measured immediately. Routine laboratory tests in both groups include procalcitonin, C-reactive protein (CRP), white blood cell count, sputum and blood cultures, nasal swab for virus multiplex PCR, *Legionella* and pneumococcal antigen in urine. Other laboratory markers as well as genetic markers will be measured in batch analysis from stored serum or plasma samples, respectively, after the study is terminated.

### Data collection

Baseline data are collected by electronic case report forms (eCRF) compliant to good clinical practice (GCP) and contain date and time of randomization, birth date, sex, medical history items (dyspnea at rest or after having taken a flight of stairs, cough, smoking history (pack-years) and status (pack per day), relevant co-morbidities, degree of autonomy at home, clinical items of pneumonia, pneumonia severity index (PSI), extent of pneumonia, underlying chronic obstructive pulmonary disease (COPD) as assessed by patient history and medical documents.

In both groups, hospitalized patients are reassessed clinically every 12 hours (including vital signs) and blood sampling on days 3, 5 and 7 including 4 measurements of glucose levels on days 3, 5 and 7, respectively. Details on dosing of all prescribed antimicrobials during the study period are recorded. In hospitalized patients, hospital discharge is recommended if oral intake is feasible, vital signs are stable ≥ 24 hours, and no evidence of acute serious co-morbidity that necessitates hospitalization is found. A final blood collection is foreseen on the day of discharge.

On days 1 and 5, the patient has to complete a short questionnaire on respiratory symptoms.

Any additional diagnostic testing in patients in both groups is at the full discretion of the treating physician.

### Follow-up

Structured follow-up telephone interviews for secondary outcomes after discharge are performed on days 30 and 180. In case the patient cannot be contacted or cannot give sufficient information, the primary care physician is interviewed.

Follow-up items include assessment of adverse events, such as infections, recurrent pneumonia, re-hospitalization, new diabetes or insulin dependence, new hypertension, and the standardized questionnaire on respiratory symptoms that was used on days 1 and 5.

### Endpoints

#### Primary endpoint

Primary endpoint is time to clinical stability, which is defined as follows: time (days) until stable vital signs for ≥ 24 hours [21]:

Temperature ≤ 37.8°C without antipyretic agents, heart rate/minute ≤ 100, spontaneous respiratory rate ≤ 24 per minute, systolic blood pressure ≥ 90 mmHg (≥100 mmHg for patients diagnosed with hypertension) without vasopressor support, mental status back to level before CAP, adequate oxygenation on room air of oxygen therapy (PaO_2_ ≥ 60 mmHg or pulse oximetry ≥ 90%). For patients with chronic hypoxemia or chronic oxygen therapy, PaO_2_ or pulse oximetry measurement must be back to baseline. Nurses on the wards unaware of treatment allocation assess clinical stability during hospitalization every 12 hours.

#### Secondary endpoints

We will consider the following secondary endpoints:

• All-cause mortality within 30 and 180 days

• ICU stay and time to transfer to ICU

• in ICU patients: time to discharge from ICU; duration of vasopressor treatment; duration of mechanical ventilation

• Time to effective hospital discharge (days)

• Duration of intravenous and total antibiotic treatment (days)

• Disease activity scores. This will be evaluated by a CAP specific disease activity score [25]. Number of days with restriction from CAP, and daily function and health state will be evaluated, all assessed at baseline, at days 5, 30 and 180

• Side effects and adverse effects of corticosteroid treatment (which are rate of hyperglycemia, hypertension, weight gain, delirium, nosocomial infections) as assessed by a validated questionnaire [26] as well as by blood glucose, weight and blood pressure monitoring

• Incidence of CAP complications until days 30 and 180 (which are persistence of pneumonia, acute respiratory distress syndrome (ARDS), empyema)

• Microbiological, clinical and/or radiological recurrence within 30 and 180 days

• Time to earliest possible hospital discharge (days) based on objective criteria (possible oral intake of food, liquids and drugs, stable vital signs > 24 hours, recovery from CAP-related worsening of mental status, no evidence of acute serious CAP-related co-morbidity that necessitates hospitalization. This endpoint will minimize a potential bias due to extended hospitalization for non-disease specific reasons (request from patients or their relatives, lack of adequate home care support, or possibility for transferral to nursing home, lack of assurance about compliance with treatment)

• Late failure (that is recurrence of signs and symptoms of pneumonia after 72 hours of randomization after an initially beneficial response to treatment) [12]

### Subgroup analyses

We plan to explore treatment effects according to the following parameters:

• Above versus below the median initial CRP and maximal CRP (hypothesis: patients with higher CRP benefit more from adjunct corticosteroid therapy)

• Age < 70 years versus > 70 years (hypothesis: patients above 70 years benefit more, as older-aged patients present with more severe CAP and have a higher mortality [27]. So far, there is no age-related data on adjunct glucocorticoid therapy, but in severe CAP there is more evidence for benefit of glucocorticoid therapy [16])

• Severity of CAP (low-risk group: PSI I to III versus high-risk group: PSI IV to V (hypothesis: patients with more severe CAP and supposedly more severe inflammation benefit more)

• Microbiological subgroups with versus without bacteremia (hypothesis: patients with bacteremia benefit more)

• With versus without underlying COPD according to the Global initiative for chronic Obstructive Lung Disease (GOLD) classification (hypothesis: patients with COPD benefit more)

• COPD patients with GOLD category I to II versus III to IV (hypothesis: patients with GOLD category III to IV benefit more)

### Sample size considerations

This study is designed to show superiority of the steroid group compared to placebo treatment. Our primary hypothesis is that adjunct corticosteroid treatment in patients with CAP will result in a reduced time to clinical stability compared to placebo treatment. To estimate the frequency of our primary endpoint, we used data of CAP patients from our completed studies [24,28,29] which were performed in the same study centers. Based on these previous trials, we assumed a mortality rate of 10% in the placebo group and 7.5% in the corticosteroid group over 14 days of follow-up with a proportion of 75% survivors being clinically stable after 7 days in the corticosteroid group. Estimating a decrease in the risk of non-stability after 1 week among survivors by 25% through adjunct corticosteroids, a sample size of 800 patients followed for at least 14 days after randomization will be needed to achieve a statistical power of 85% and a sample size of 700 patients to achieve a statistical power of 80% (2-sided type 1 error of 5% in both scenarios). We intend to recruit 800 study patients from December 2009 to April 2014.

### Intention-to-treat and per-protocol population

Following the intention-to-treat principle, all patients receiving at least one dose of study medication will be included in the analysis with group allocation as randomized; patients violating inclusion criteria or meeting exclusion criteria due to information that was not available at study entry will be excluded post randomization in a blinded manner [30]. Criteria for exclusion of patients from the per-protocol analysis are, in addition, withdrawal of informed consent or non-compliance with study medication or the concomitant procedures. Therefore, patients stopping or pausing the study medication at any time point, be it due to corticosteroid contraindications or due to active indication for glucocorticoid treatment, will not be considered in the per-protocol analysis. A patient will not be followed up if he withdraws informed consent for all study measures; in all other cases, follow-up will be performed as planned.

### Statistical analysis

We will enter all relevant clinical and laboratory data obtained by interview, clinical tests, and reviewing of the medical records into an online database. Statistical Analysis System (SAS® Institute, Cary, NC, USA) and R for Windows (R Foundation for Statistical Computing, http://www.r-project.org) and STATA 9.2 (Stata Corp, College Station, TX, USA) will be used for data analysis.

**Figure 2:**
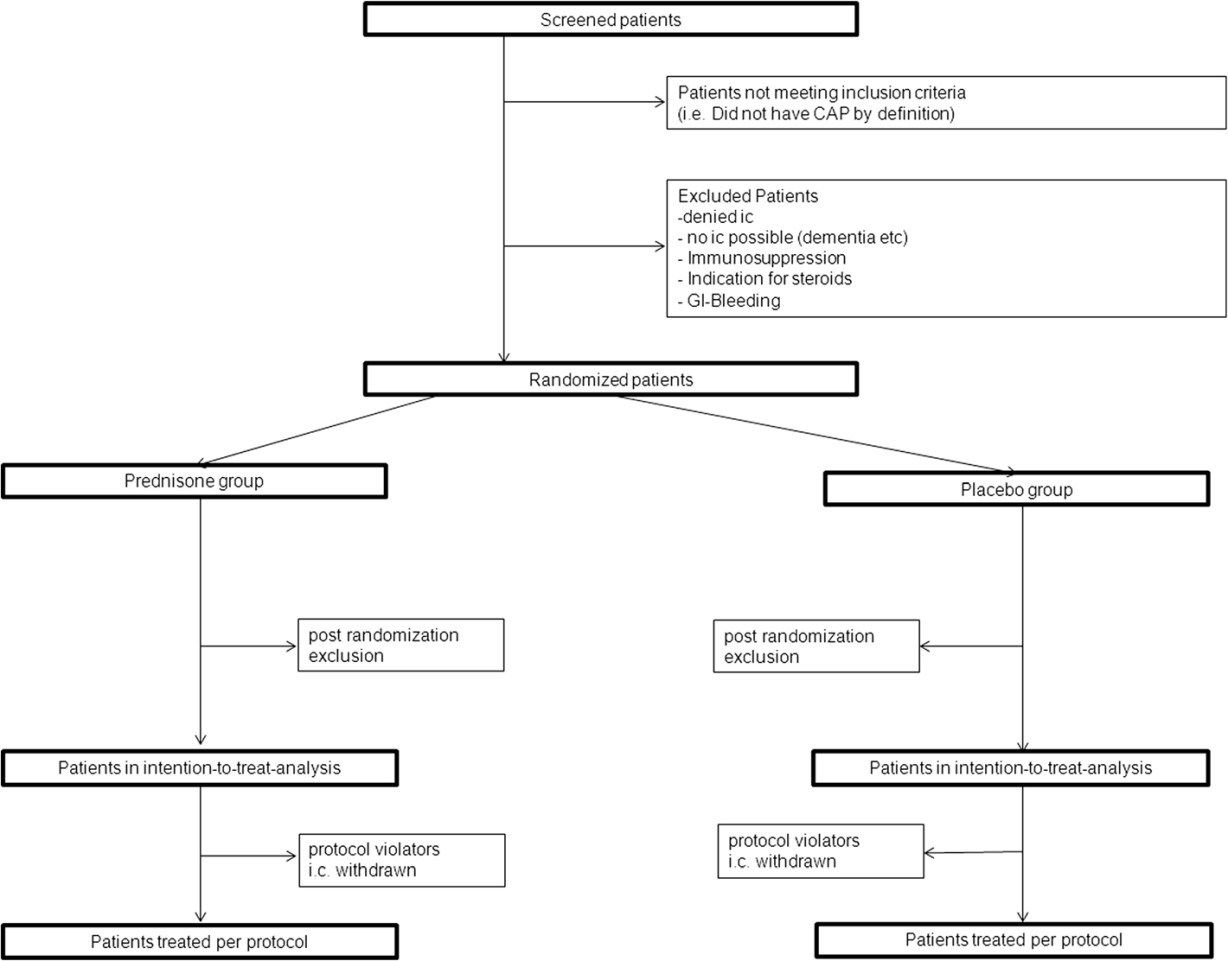
CONSORT diagram of the trial.

The primary analysis population is the full analysis set which includes the intention-to-treat population. Every effort will be made to minimize the number of losses to follow-up by recording phone numbers before discharge, thereafter by repetitive follow-up calls by home or cell phone contact, or alternatively by contacting the primary care physician. For the primary outcome of time to clinical stability, we consider losses to follow-up in this sample of hospitalized patients as unlikely. If losses to follow-up do occur, patients will be censored at time of last assessment for clinical stability. A secondary analysis population, the per-protocol population, will exclude patients not treated according to the protocol (that is protocol violators). For the primary endpoint, we will calculate an unadjusted hazard ratio and 95% confidence interval by means of a Cox proportional hazards model. The primary objective is to show superiority of the corticosteroid group at a 2-sided 5% alpha-level with 85% power following the intention-to-treat principle. As a sensitivity analysis, the primary analysis will be repeated on the per-protocol patient population. As a further sensitivity analysis, a multivariable Cox proportional hazards model will be fitted with treatment group, patient age, and PSI scores as independent variables. This model will adjust the treatment effect for the potentially important confounders age and PSI score. Moreover, we plan to investigate whether the treatment effect varies among different degrees of severity of CAP (low-risk group: PSI I to III, and high-risk group: PSI IV to V), between patients with and without underlying COPD, and between bacteremic versus non-bacteremic patients. COPD will be defined by post-bronchodilator spirometric criteria, according to the GOLD guidelines [31], as a FEV1/FVC ratio below 70% and the severity categorized according to the GOLD criteria. In patients with a clinical history of COPD or smoking, lung function at the time of inclusion will not be mandatory. We will conduct the pre-specified subgroup analyses by including appropriate interaction terms in the above described multivariable Cox proportional hazards model.Complete case analyses will be used for secondary endpoints. For all secondary endpoints, we will calculate unadjusted and adjusted estimates of the effect size and corresponding 95% confidence intervals using linear, logistic, or Cox proportional hazards regression (as appropriate). Figure 2 shows the CONSORT diagram of the trial.

### Study organization

All adverse events of category III or more according to Common Terminology Criteria for Adverse Events v4.0 [32] are recorded. Adverse events will be monitored for every subject participating in the study and attributed to the study medication and reported to the respective authorities according to law if necessary.

The trial is supervised by an independent and blinded DSMB that is not involved in the design and conduct of the trial, and has no affiliation with the sponsor. The DSMB is comprised of experts from different disciplines and supervises the recruitment of patients, patient flow and patient interviews. The board has access to the randomization code in case of need. In case of serious problems or adverse events that may occur during the trial, the board will review the preplanned interim analysis and inform the principle investigators whether the trial may be continued or has to be stopped early or it may require modifications of the protocol.

A blinded safety interim analysis was performed after 50% of patients had completed the 180 day follow-up. The DSMB recommended continuation of the study without changes to the protocol.

In accordance with applicable regulations and GCP, a study monitor is periodically controlling study procedures.

### Planned subprojects

We will use data collected during this trial for further analyses investigating the following topics:

• Diagnostic value of adrenal function testing (ACTH test)

• Prognostic value of hormonal and inflammatory biomarkers (for example, total and free cortisol levels, copeptin, dehydroepiandrosterone (DHEA) as well as the ratio of cortisol to DHEA [33]) in a further attempt to identify those patients who benefit most from corticosteroid treatment

• Effect of corticosteroid treatment on inflammatory markers

• Prognostic value of hormonal biomarkers for risk assessment in patients with CAP

• Microbiological etiology of pneumonia (bacterial testing in blood, sputum and urine, viral multiplex PCR from nasal swabs)

• Predictability of patients and doctors whether the study medication was prednisone or placebo

## Discussion

Even with the prescription of newer and more potent antimicrobial agents, mortality due to CAP has remained relatively constant over time. Corticosteroids have been proved to block several arms of the inflammatory cascade. Available evidence so far, derived from a few, mainly small randomized controlled trials, suggests that corticosteroid treatment might be beneficial in terms of survival in patients with CAP [6,9,10,34-36].

Most studies showing a beneficial effect of corticosteroids in patients with CAP only included severe pneumonia, as defined with a PSI class IV and V [11,34,37,38]. In these patients, reviews and meta-analyses [13-16] pointed to a possible benefit of corticosteroids on either length of stay or mortality without evidence of more complications. However, insufficient data on non-severe CAP are available. Recently, two randomized double-blind controlled trials including 200 to 300 patients with all severities of CAP found controversial results: the first study using 40 mg of prednisolone for seven days showed no effect on time to clinical stability, with a higher rate of late failure in the corticosteroid group, defined by recurrence of signs and symptoms of pneumonia after 72 hours of admission after an initially beneficial response to treatment [12]. The second study using 4 days of 5 mg of dexamethasone intravenously showed a significantly shorter duration of hospital stay in patients treated with corticosteroids [6,12]. In view of these controversial findings, a large prospective and adequately powered trial should conclusively determine the risks and benefits of adding corticosteroids to the treatment of patients with CAP.

In the current trial, we will include all severity levels of CAP (PSI I to V) requiring hospitalization. This will lead to a lower mortality rate than observed in previous studies only including severe CAP patients, and our study will not be powered to show a survival benefit. We therefore chose as primary endpoint time to clinical stability, which is a less stringent but still clinically relevant endpoint. By reducing the time to clinical stability and consecutively also length of stay (LOS), nosocomial complications like infections, thrombembolic events, worsening of pre-existing frailty or delirium may be prevented. This leads to better allocation of resources at the hospital and to a marked reduction of costs [39]. While Meijvis *et al*. looked at LOS [6], our primary endpoint will be time to clinical stability, as defined by IDSA/ATS guidelines [22]. As we have previously observed, LOS itself in community-acquired pneumonia may be confounded and prolonged by medical or organizational problems unrelated to CAP [39-41]. We believe that by measuring time to clinical stability, we will be looking at a clinically relevant parameter, as patients are able to switch to oral antibiotics and ready for discharge once they reach stability.

Inclusion of all patients with CAP will increase the generalizability of the findings and allow retrospective evaluation in exploratory analyses as to which patients indeed profited most from corticosteroid treatment.

In our study, we administer an oral dose of 50 mg prednisone for 7 days without tapering. We opted for an oral formula, as the bioavailability of oral prednisone is excellent, it may be ground and is therefore applicable in patients not able to swallow, such as in patients on mechanical ventilation or older-aged patients. Furthermore, by choosing a uniform application formula, further bias, like the ability to blind and to secure provision of blinded study medication 24 hours on 7 days per week, was prevented.

The ideal choice and duration of corticosteroid treatment in critically ill patients and patients with CAP is currently disputed. Hydrocortisone is the natural corticosteroid and was used in some studies in critically ill patients, partly together with a mineralocorticoid [42]. Most patients in our study, however, will not suffer from critical illness and will be hospitalized outside the ICU on the wards where oral intake of treatment is preferred. Since hydrocortisone is only available in 10 mg tablets, patients would have to take 20 tablets a day, which does not appear feasible. Methylprednisolone and prednisolone have also been used in some studies including patients with CAP and ARDS [10,12,43,44], putting it forward as an alternative formulation for our study, as adequate oral formulations are available. Prednisolone is the pharmacologically active metabolite of prednisone, which was used in this study; both are used interchangeably except in patients with severe liver insufficiency, where prednisolone is preferred. Dexamethasone was not recommended by guidelines for use in septic shock and ARDS at the time of study design [37]. However, a recent study showed a reduced length of hospital stay in CAP patients treated with dexamethasone as compared to placebo [6], also putting it forward as a possible type of corticosteroid in patients with CAP. The additional use of fludrocortisone as in the study by Annane *et al*. [42] is currently considered optional [37]. Furthermore, the optimal duration of glucocorticoid treatment and mode of discontinuing (tapering or not) is not known and heavily disputed [45,46].

In high-dose glucocorticoid studies, which used as high as 1 to 3 g hydrocortisone per day, short-term complications, such as co- and re-infections, led to excess mortality [47,48]. In so-called low- or physiological-dose glucocorticoid studies with 100 to 300 mg hydrocortisone-equivalent per day, the main adverse effects included hyperglycemia [6] and rebound pneumonia [12]. Although confusion, co-infection and gastrointestinal side effects were reported in association with glucocorticoid treatment, the incidence was not significantly higher in the steroid group [16]. While some authors advise five days of glucocorticoid treatment in critically ill patients without tapering [49-51], others recommend tapering of glucocorticoids to avoid a rebound of inflammatory markers with consecutive rebound pneumonia [37,45,46,52]. However, it has been shown that glucocorticoids given > seven days lead to a worse clinical course measured by length of stay, clinical stability and mechanical ventilation, and more systemic complications when looking at shock and cardiac arrhythmia [53]. From COPD studies, there is evidence that stopping glucocorticoids without tapering is safe without an increased recurrence rate [54]. For these reasons, we decided against tapering of the glucocorticoid dose in order not to prolong glucocorticoid exposure.

In conclusion, this current large and adequately powered randomized trial will determine the risks and benefits of adding 50 mg of prednisone for 7 days to the treatment of hospitalized patients with CAP. Our hypothesis is that corticosteroid treatment will result in a reduced time to clinical stability in CAP compared to placebo treatment.

### Study limitations

The results of this trial may only be valid for the specific type, dose and duration of glucocorticoid studied.

This study is powered for clinical stability, but not for mortality; at least 4,000 patients would be needed to be powered to detect a clinically relevant difference in mortality.

Furthermore, we also included lower severity CAP patients, which might weaken the effect of glucocorticoids. A conclusion for ICU patients might not be possible as the number of these patients included will be low. There is an ongoing ICU multicenter study (clinicaltrials.gov number NCT01448109) aiming for the goal of 4,000 patients that will hopefully answer this question for ICU patients with CAP.

## Trial status

Recruiting. On 15 October 2013, 670 patients had been enrolled in the study.

## Abbreviations

ACTH: adrenocorticotropic hormone; ARDS: acute respiratory distress syndrome; CAP: community-acquired pneumonia; CONSORT: Consolidated Standards of Reporting Trials; COPD: chronic obstructive pulmonary disease; CRP: C-reactive protein; DHEA: dehydroepiandrosterone; DSMB: Data Safety and Monitoring Board; eCRF: electronic case report form; GCP: good clinical practice; GOLD: The Global initiative for chronic Obstructive Lung Disease; PCR: polymerase chain reaction; PSI: Pneumonia Severity Index; STEP: ‘Corticosteroid treatment for community-acquired pneumonia’ trial.

## Competing interests

The authors declare that they have no competing interests with regards to this study.

## Authors’ contributions

CAB was involved in the study design and writing of the protocol, study coordination, recruited patients for the study, participated in statistical analysis and drafted the manuscript. MB participated in designing the study and writing the protocol, did the statistical analysis and helped draft the manuscript. IS was involved in the study design, study coordination and recruited patients for the study. NN was involved in study coordination and recruited patients for the study. BW was involved in study coordination and recruited patients for the study. RB was involved in study coordination and gave financial and staff support. WZ was involved in study coordination and gave financial and staff support. EU participated in study coordination and recruited patients for the study. HE participated in study coordination and recruited patients for the study. PT was involved in study coordination and gave staff support. SW was involved in study coordination and recruited patients for the study. RT was involved in study coordination and recruited patients for the study. EH was involved in study coordination and recruited patients for the study. NR was involved in study coordination, gave financial and staff support, and critically revised the manuscript. HD was involved in study coordination, gave staff support and recruited patients for the study. DB gave financial and staff support for performing the nasopharyngeal PCR. BM participated in designing the study and writing the protocol, in study coordination and gave financial and staff support. PS participated in designing the study and writing the protocol, did the statistical analysis and helped draft the manuscript. MCC designed the study, wrote the protocol, coordinated the study and gave financial and staff support. All authors read and approved the final protocol manuscript.
